# Health behavior and mental health by gender in Korean adults before, during, and after the COVID-19 pandemic

**DOI:** 10.1371/journal.pone.0325895

**Published:** 2025-06-26

**Authors:** Jae Yeon Jeong, Yoo Mi Jeong

**Affiliations:** 1 Department of Health Management, Jeonju University, Jeonju-si, Jeollabuk-do, Republic of Korea; 2 College of Nursing #206, Dan kook University, Cheonan-si, Chungnam-do, Republic of Korea; University of Jyvaskyla, FINLAND

## Abstract

**Objective:**

Coronavirus disease 2019 (COVID-19) has been a global health crisis, and its aftermath has had a profound impact on mental health and health behaviors of Korean adults. This study aimed to determine the health behavior and mental health among adults aged 19 years and older before, during, and after the COVID-19 pandemic.

**Methods:**

We used 2019–2022 data from Korea National Health and Nutrition Examination Survey (KNHANES) to analyze a comparative assessment of drinking, smoking, sleep, and stress in 2019, 2020, 2021, and 2022. Chi-square, one-way ANOVA and logistic regression analysis was performed with using Stata version 18.5.

**Results:**

The proportion of drinking more than four times a week was slightly lower in the early- and with-COVID-19 and higher in the pre- and post-COVID-19, and was the same in the analysis of gender. The proportion of smoking in men decreased over time, whereas that in women increased. Both gender increased weekday sleep time in the pre- and early-COVID-19, and decreased in the post-COVID-19. Stress was slightly reduced in the pre- and post-COVID-19 compared to early-COVID-19 in men, and has decreased in the post-COVID-19 in women. Comparing these variables adjusting for the sociodemographic variables by years, frequency of drinking decreased in 2020 and 2021 for both men and women, but there was no difference in 2022. There was no statistically significant difference in current smoking for both men and women. Suicidal ideation decreased in men in 2022, but increased in women during the early-COVID-19.

**Conclusions:**

Drinking and smoking showed a somewhat positive pattern of behavior change, but in the post-COVID-19, unhealthy behaviors have increased again, requiring active health interventions.

## 1. Introduction

The Coronavirus disease 2019 (COVID-19) pandemic has been a global health crisis, and its aftermath has had a profound impact on mental health and health behaviors of Korean adults. During the pandemic, social isolation measures and bans on gatherings of more than four people have significantly restricted economic activities and social interactions, which have particularly increased psychological anxiety and stress [1–3]. This psychological anxiety leads to anxiety and depressive symptoms, and as a result, addictive health behaviors such as drinking and smoking tend to increase [4,5]. In particular, mental health and health behavior changes appear differently depending on gender, suggesting that the pandemic has had a different impact on men and women [6].

While there have been many studies on mental health and health behaviors before and after COVID-19, most studies have relied on early pandemic data, which limits their ability to fully identify long-term changes or gender differences [7]. In particular, in the first year of 2020, stress, anxiety, and depressive symptoms increased significantly, and health behaviors such as drinking and smoking were also reported to increase in both men and women [4,8]. However, there is a lack of research on whether these changes continue after the pandemic. Monitoring gender differences in mental health and health behaviors, as well as the ongoing impact of the pandemic on men and women, is important to inform future health policies and interventions.

Therefore, this study aims to analyze the changes in mental health and health behaviors before, during, and after COVID-19 among Korean adult men and women, according to gender. Through this, we can contribute to a clear understanding of the impact of the pandemic on the health of Korean adult men and women from a gender perspective, and to suggest health maintenance and management measures after future infectious diseases according to gender.

## 2. Methods

### 2.1. Sample and data collection

We used 2019−2022 data from KNHANES, which is conducted by the Korea Disease Control and Prevention Agency (KDCA) to analysis a comparative assessment of health behaviors across distinct phases of the COVID-19 pandemic: pre-COVID-19 phase in 2019(N = 5,875), the early- COVID-19 phase in 2020 (N = 5,379), the with-COVID-19 phase in 2021(N = 5,277), and the post-COVID-19 phase in 2022(N = 4,844).

The National Health and Nutrition Examination Survey conducts a survey of about 10,000 household members over the age of 1 by sampling 4,800 households in 192 regions every year. The survey adopts a rolling sampling survey method, which is structured to represent the whole country using three independent circular samples each year. The survey is divided into children (1–11 years old), adolescents (12–18 years old), and adults (19 years old and older), and different survey items are applied to each age group. The main survey items include a medical examination that measures health status, a health questionnaire that includes subjective answers about health behaviors, and a nutrition survey. This study used data from surveys of adults aged 19 years and older. We used the Stata/MP version 18.5, chi-square analysis, one-way ANOVA and logistic regression analysis was performed. Through this, differences in health behaviors and mental health changes by gender and year before and after the COVID-19 pandemic were statistically analyzed.

### 2.2. Ethics and data gathering procedure

The KNHANES raw data is publicly accessible and written informed consent was obtained from the survey participants. The KNHANES raw data has been anonymized and processed in a manner that prevents the identification of research subjects. It is publicly available for scientific purpose, and we accessed the KNHANES for research purposes on April 24, 2024, via https://chs.kdca.go.kr/. The data consequently, researchers have no access to any personally identifiable information.

## 3. Measures

### 3.1. Variables

#### 3.1.1. Sociodemographic variables.

The sociodemographic variables used in this study are gender, age, household income, educational level, type of residence, and region of residence. Educational attainment was categorized as elementary school graduation or less, high school graduation, and college graduation, and household income was divided into quintile. The type of residence was classified according to whether they lived with others or alone, and the area of residence was classified into the metropolitan area, Daegu city, and the province. Daegu City was an area where a cluster of rapid community outbreaks occurred during the initial COVID-19 pandemic in 2020, and the government implemented a policy to isolate the Daegu area [9]. Given the distinct outbreak dynamics and governmental response measures in Daegu, we analyzed this region separately to better capture localized variations in health behaviors and mental health outcomes. Although the separation may have slightly reduced the overall sample size, it allowed for a more nuanced understanding of regional effects without significantly compromising the generalizability of the findings, as Daegu comprised only a small proportion of the national sample. This regional focus provides valuable insights into how intense local outbreaks can differentially impact public health trends.

#### 3.1.2. Drinking behavior.

Drinking behavior variables include frequency of drinking, amount of alcohol consumed, frequency of binge drinking, and drinking problems over a one-year period. The frequency of drinking was divided into ‘not drinking at all’, ‘less than 1 drink per month’, ‘1 time per month’, ‘2-4 times a month’, ‘2-3 times a week’, and ‘more than 4 times a week’, and the amount of alcohol consumed was divided into ‘not drinking at all’, ‘1-2 drinks’, ‘3-4 drinks’, ‘5-6 drinks’, ‘7-9 drinks’, and ‘10 or more drinks’. The Korea Disease Control and Prevention Agency recommends drinking no more than four drinks a day and no more than twice a week as an appropriate amount of alcohol. The frequency of binge drinking was divided into ‘never’, ‘less than 1 time per month’, ‘1 time per month’, ‘1 time per week’, and ‘almost every day’, and the drinking problem was classified as ‘present’ and ‘none’.

#### 3.1.3. Smoking behavior.

Smoking behavior variables include recent smoking, daily smoking, e-cigarette smoking, and daily e-cigarette smoking. Recent smoking and e-cigarette smoking were divided into ‘never’, ‘daily’, ‘occasionally’, and ‘ex-smokers’, and the amount of daily smoking and daily e-cigarette smoking were measured as continuous variables. In addition, the frequency of these activities before and after the pandemic was categorized into increase, no change, and decrease.

#### 3.1.4. Sleep and mental health.

Sleep and mental health were categorized according to changes in daily life. The study compared changes in physical activity, consumption of convenience foods, alcohol consumption, smoking, household income, and work environment before and after the pandemic, and categorized their frequency as increasing, unchanged, or decreasing compared to pre-pandemic levels. Variables related to mental health included major psychological changes such as stress, anxiety, and depression. To comprehensively assess mental health status during the COVID-19 pandemic, additional indicators such as suicidal ideation, suicide attempts, and psychological counseling were also included. This multidimensional approach allowed for a more understanding of mental health outcomes in the context of pandemic-related life changes.

### 3.2. Statistical analysis

Data were analyzed using Stata version 18.5 for Windows. A two-sided p-value of <.05 was considered significant. Frequencies and percentages were calculated for the characteristics of the men and women groups. To compare health behaviors before, during, and after the COVID-19 pandemic, chi-square analysis was performed. For continuous variables such as daily cigarette consumption and sleep duration, one-way analysis of variance (ANOVA) followed by Scheffé’s post hoc test was conducted to determine inter-year differences, In addition, logistic regression analyses were performed using binary outcome variables (smoking: yes/no, drinking: yes/no, suicidal ideation: yes/no), adjusting for sociodemographic covariates to explore trends over time, and 95% confidence intervals (CIs) were obtained.

## 4. Results

### 4.1. Socio-demographic, health and health behavioral characteristics of participants

Table 1 shows the sociodemographic characteristics of the study participants. The proportion of women compared to men was slightly higher, and there was no statistically significant difference between before and after COVID-19. In terms of age, the proportion of people in their 40s ~ 60s was the highest, and the difference between years is statistically significant (p < 0.001). Income quintiles were divided according to the baseline amount of average personal income by gender and age classified into quintiles, and there was no significant difference. In terms of education level, college graduates and above were the most numerous, followed by high school graduates, elementary school graduates and juniors, and junior high school graduates. In the case of the type of residence, it was confirmed that the proportion of single-person households increased over time (13.0% ~ 16.1%), and there was a statistically significant difference. The proportion of residents living in the Daegu area was as low as about 4%, and the ratio between the metropolitan area and the provinces was similar.

**Table 1:** General characteristics of the participants categorized by the respective year.

Variable		2019(n = 5,875)		2020(n = 5,379)		2021(n = 5,277)		2022(n = 4,844)		*P*-value
Sex	Male	2594	(44.1)	2427	(45.1)	2329	(44.1)	2091	(43.1)	0.267
	Female	3281	(55.8)	2952	(54.8)	2948	(55.8)	2753	(56.8)	
Age	19-29	718	(12.2)	774	(14.3)	657	(12.4)	584	(12.0)	0.000
	30-39	880	(14.9)	732	(13.6)	622	(11.7)	611	(12.6)	
	40-49	1072	(18.2)	914	(16.9)	910	(17.2)	800	(16.5)	
	50-59	1125	(19.1)	1010	(18.7)	964	(18.2)	836	(17.2)	
	60-69	1058	(18.0)	1045	(19.4)	1047	(19.8)	1057	(21.8)	
	≥ 70	1022	(17.4)	904	(16.8)	1077	(20.4)	956	(19.7)	
Income	Q1	1151	(19.5)	1030	(19.1)	1041	(19.7)	963	(19.8)	0.999
	Q2	1161	(19.7)	1075	(19.9)	1029	(19.5)	970	(20.0)	
	Q3	1167	(19.8)	1097	(20.3)	1065	(20.1)	955	(19.7)	
	Q4	1201	(20.4)	1089	(20.2)	1080	(20.4)	973	(20.0)	
	Q5	1195	(20.3)	1088	(20.2)	1062	(20.1)	983	(20.2)	
education	Elementary school	1068	(18.1)	904	(16.8)	993	(18.8)	883	(18.2)	0.002
	Middle school	556	(9.46)	522	(9.7)	535	(10.1)	412	(8.51)	
	High school	1965	(33.4)	1894	(35.2)	1781	(33.7)	1586	(32.7)	
	≥ College	2286	(38.9)	2059	(38.2)	1968	(37.2)	1963	(40.5)	
Residence type	alone	769	(13.0)	701	(13.0)	836	(15.8)	781	(16.1)	0.000
	with other	5106	(86.9)	4678	(86.9)	4441	(84.1)	4063	(83.8)	
Region	Capital	2657	(45.2)	2483	(46.1)	2278	(43.1)	2131	(43.9)	0.000
	Daegu-si	285	(4.85)	169	(3.14)	222	(4.21)	197	(4.07)	
	Province	2933	(49.9)	2727	(50.7)	2777	(52.6)	2516	(51.9)	

n (%), the χ2 test has been performed.

### 4.2. Drinking behaviors

In this study, the proportion of respondents who said they had not drunk alcohol for one year increased significantly in the early COVID-19 period and with COVID compared to the pre-COVID period, but decreased in the post-COVID period (Table 2). The proportion of women who did not drink alcohol for 1 year was about 2 ~ 3 times higher than that of men, and the pattern of increasing the proportion before and after the pandemic was the same in the analysis of gender (Tables 3 and 4). The proportion of drinking more than four times a week was slightly lower in the early COVID and with COVID periods, and higher in the pre- and post-COVID periods (Table 2), and was the same in the analysis of gender. In terms of the amount of alcohol consumed, the proportion of 3 ~ 4, 5 ~ 6 drinks in both men and women decreased, and the proportion of those who drank more than 10 drinks increased. For men, the proportion of 1 ~ 2 cups was higher, and for women, the proportion of 7 ~ 9 cups was higher, showing a difference. Binge drinking rates were higher in absolute numbers for men than for women, but in the time-wise variation, they gradually decreased for men and increased for women in groups almost daily. Drinking problems decreased during the pandemic but increased again in the post-COVID period, with the same results in the gender analysis (Tables 3 and 4).

**Table 2:** Drinking Behaviors of the participants categorized by the respective year.

Variable		2019(n = 5,875)		2020(n = 5,379)		2021(n = 5,277)		2022(n = 4,844)		*P*-value
Frequency of drinking	Never	1631	(27.7)	1604	(29.8)	1731	(32.8)	1439	(29.7)	0.000
over a year	Less than once a month	1066	(18.1)	1003	(18.6)	972	(18.4)	971	(20.0)	
	Once a month	663	(11.2)	545	(10.1)	500	(9.5)	461	(9.5)	
	2-4 times a month	1278	(21.7)	1137	(21.1)	1099	(20.8)	999	(20.6)	
	2-3 times a week	860	(14.6)	793	(14.7)	662	(12.5)	680	(14.0)	
	More than 4 times a week	377	(6.42)	297	(5.52)	313	(5.93)	294	(6.07)	
Amount of alcohol	Never	1631	(27.7)	1604	(29.8)	1731	(32.8)	1439	(29.7)	0.000
consumed at one time	1-2 glasses	1520	(25.8)	1360	(25.2)	1321	(25.0)	1285	(26.5)	
	3-4 glasses	898	(15.2)	764	(14.2)	715	(13.5)	671	(13.8)	
	5-6 glasses	604	(10.2)	481	(8.94)	442	(8.38)	361	(7.45)	
	7-9 glasses	650	(11.0)	607	(11.2)	588	(11.1)	574	(11.8)	
	10 glasses more	572	(9.7)	563	(10.4)	480	(9.1)	514	(10.6)	
Frequency of binge drinking	Never	1921	(32.6)	1845	(34.3)	1946	(36.9)	1625	(33.5)	0.000
	Less than once a month	1460	(24.8)	1277	(23.7)	1352	(25.6)	1311	(27.0)	
	Once a month	909	(15.4)	804	(14.9)	719	(13.6)	662	(13.6)	
	Once a week	786	(13.3)	695	(12.9)	559	(10.5)	560	(11.5)	
	almost every day	799	(13.6)	758	(14.0)	701	(13.2)	686	(14.1)	
Drinking Problems: Noisiness	Yes	387	(6.6)	378	(7.0)	264	(5.0)	315	(6.5)	0.000
	No	5488	(93.4)	5001	(92.9)	5013	(95.0)	4529	(93.5)	
Drinking Problems: Drunk Driving	Yes	33	(0.56)	8	(0.15)	11	(0.21)	14	(0.29)	0.000
	No	5842	(99.4)	5371	(99.8)	5266	(99.7)	4830	(99.7)	

n (%), the χ2 test has been performed.

**Table 3:** Drinking Behaviors of the male participants categorized by the respective year.

Variable		2019(n = 2,594)		2020(n = 2,427)		2021(n = 2,329)		2022(n = 2,091)		*P*-value
Frequency of drinking	Never	443	(17.0)	478	(19.7)	531	(22.8)	416	(19.8)	0.000
over a year	Less than once a month	306	(11.8)	333	(13.7)	302	(12.9)	317	(15.1)	
	Once a month	284	(10.9)	233	(9.6)	218	(9.4)	189	(9.0)	
	2-4 times a month	695	(26.7)	598	(24.6)	577	(24.7)	509	(24.3)	
	2-3 times a week	555	(21.4)	550	(22.6)	442	(18.9)	431	(20.6)	
	More than 4 times a week	311	(11.9)	235	(9.7)	259	(11.1)	229	(10.9)	
Amount of alcohol	Never	443	(17.0)	478	(19.7)	531	(22.8)	416	(19.8)	0.000
consumed at one time	1-2 glasses	394	(15.1)	387	(15.9)	382	(16.4)	356	(17.0)	
	3-4 glasses	447	(17.2)	372	(15.3)	338	(14.5)	329	(15.7)	
	5-6 glasses	368	(14.1)	305	(12.5)	271	(11.6)	196	(9.37)	
	7-9 glasses	495	(19.0)	429	(17.6)	434	(18.6)	412	(19.7)	
	10 glasses more	447	(17.2)	456	(18.7)	373	(16.0)	382	(18.2)	
Frequency of binge drinking	Never	673	(25.9)	677	(27.9)	642	(27.5)	566	(27.0)	0.000
	Less than once a month	444	(17.1)	435	(17.9)	461	(19.7)	440	(21.0)	
	Once a month	433	(16.6)	366	(15.0)	352	(15.1)	318	(15.2)	
	Once a week	467	(18.0)	413	(17.0)	320	(13.7)	300	(14.3)	
	almost every day	577	(22.2)	536	(22.0)	484	(20.7)	467	(22.3)	
Drinking Problems: Noisiness	Yes	193	(7.4)	185	(7.6)	131	(5.6)	152	(7.3)	0.027
	No	2401	(92.5)	2242	(92.3)	2198	(94.3)	1939	(92.7)	
Drinking Problems: Drunk Driving	Yes	23	(0.89)	7	(0.29)	10	(0.43)	9	(0.43)	0.019
	No	2571	(99.1)	2420	(99.7)	2319	(99.5)	2082	(99.5)	

n (%), the χ2 test has been performed.

**Table 4:** Drinking Behaviors of the female participants categorized by the respective year.

Variable		2019(n = 3,281)		2020(n = 2,952)		2021(n = 2,948)		2022(n = 2,753)		*P*-value
Frequency of drinking	Never	1188	(36.2)	1126	(38.1)	1200	(40.7)	1023	(37.1)	0.033
over a year	Less than once a month	760	(23.1)	670	(22.7)	670	(22.7)	654	(23.7)	
	Once a month	379	(11.5)	312	(10.5)	282	(9.6)	272	(9.9)	
	2-4 times a month	583	(17.7)	539	(18.2)	522	(17.7)	490	(17.8)	
	2-3 times a week	305	(9.3)	243	(8.23)	220	(7.46)	249	(9.04)	
	More than 4 times a week	66	(2.0)	62	(2.1)	54	(1.8)	65	(2.4)	
Amount of alcohol	Never	1188	(36.2)	1126	(38.1)	1200	(40.7)	1023	(37.1)	0.004
consumed at one time	1-2 glasses	1126	(34.3)	973	(32.9)	939	(31.8)	929	(33.7)	
	3-4 glasses	451	(13.7)	392	(13.2)	377	(12.7)	342	(12.4)	
	5-6 glasses	236	(7.2)	176	(6.0)	171	(5.8)	165	(6.0)	
	7-9 glasses	155	(4.7)	178	(6.0)	154	(5.2)	162	(5.9)	
	10 glasses more	125	(3.8)	107	(3.6)	107	(3.6)	132	(4.8)	
Frequency of binge drinking	Never	1248	(38.0)	1168	(39.5)	1234	(41.9)	1059	(38.4)	0.001
	Less than once a month	1016	(30.9)	842	(28.5)	891	(30.2)	871	(31.6)	
	Once a month	476	(14.5)	438	(14.8)	367	(12.4)	344	(12.5)	
	Once a week	319	(9.7)	282	(9.6)	239	(8.1)	260	(9.4)	
	almost every day	222	(6.8)	222	(7.5)	217	(7.4)	219	(8.0)	
Drinking Problems: Noisiness	Yes	194	(5.9)	193	(6.5)	133	(4.5)	163	(5.9)	0.007
	No	3087	(94.0)	2759	(93.4)	2815	(95.4)	2590	(94.0)	
Drinking Problems: Drunk Driving	Yes	10	(0.3)	1	(0.03)	1	(0.03)	5	(0.18)	0.010
	No	3271	(99.7)	2951	(99.9)	2947	(99.9)	2748	(99.8)	

n (%), the χ2 test has been performed.

### 4.3. Smoking behavior

Among men, the number of people who said they did not smoke increased during the COVID-19 period and again to 24.6% in the post-COVID period. In contrast, there was no statistically significant difference in the proportion of non-smokers among women. For men, the proportion decreased over time in both the daily and occasional smoking groups, but there was no significant change in the proportion for women. There was no statistically significant difference in the amount of smoking in men, but in women, there was a significant decrease in smoking from 8.9 in 2019 to 7.7 in 2020, but it increased again to 8.29 in 2021 and 9.90 in 2022, which is higher than in the pre-pandemic period (Table 5).

**Table 5:** Smoking Behaviors of the participants categorized by the respective year.

Variable		2019(n = 5,875)		2020(n = 5,379)		2021(n = 5,277)		2022(n = 4,844)		*P*-value
Current smoking	Never	3504	(59.6)	3220	(59.8)	3188	(60.4)	2955	(61.0)	0.020
	Everyday	863	(14.6)	774	(14.3)	725	(13.7)	638	(13.1)	
	Sometimes	153	(2.6)	134	(2.5)	108	(2.1)	83	(1.71)	
	Past smoker	1355	(23.0)	1251	(23.2)	1256	(23.8)	1168	(24.1)	
Cigarettes per day	Never	4859	(82.7)	4471	(83.1)	4444	(84.2)	4123	(85.1)	
	(continuous)	12.82	±7.00	12.55	±7.67	12.93	±7.93	12.86	±7.29	0.724
Current smoking: electronic cigarettes	Never	5345	(90.9)	4956	(92.1)	4856	(92.0)	4440	(91.6)	0.000
	Everyday	151	(2.6)	129	(2.4)	115	(2.2)	143	(3.0)	
	Sometimes	88	(1.5)	34	(0.6)	35	(0.7)	40	(0.83)	
	Past smoker	291	(5.0)	260	(4.8)	271	(5.1)	221	(4.6)	
Electronic cigarettes per day	Never	5345	(90.9)	4956	(92.1)	4856	(92.0)	4440	(91.6)	
	(continuous)	9.66	±6.94	10.30	±6.53	9.47	±6.73	10.05	±6.34	0.667
Male										
Current smoking	Never	625	(24.0)	624	(25.7)	601	(25.8)	515	(24.6)	0.018
	Everyday	734	(28.3)	659	(27.1)	608	(26.1)	532	(25.4)	
	Sometimes	111	(4.3)	93	(3.8)	69	(3.0)	61	(2.9)	
	Past smoker	1124	(43.3)	1051	(43.3)	1051	(45.1)	983	(47.0)	
Cigarettes per day	Never	1749	(67.4)	1675	(69.0)	1652	(70.9)	1498	(71.6)	
	(continuous)	13.63	±6.96	13.57	±7.70	14.00	±7.90	13.51	±7.40	0.622
Current smoking: electronic cigarettes	Never	2154	(83.0)	2080	(85.7)	1983	(85.1)	1772	(84.7)	0.000
	Everyday	131	(5.1)	108	(4.5)	92	(4.0)	114	(5.5)	
	Sometimes	66	(2.5)	29	(1.2)	28	(1.2)	28	(1.3)	
	Past smoker	243	(9.4)	210	(8.7)	226	(9.7)	177	(8.5)	
Electronic cigarettes per day	Never	2154	(83.0)	2080	(85.7)	1983	(85.1)	1772	(84.7)	
	(continuous)	10.36	±7.19	10.5	±6.23	10.04	±7.09	10.6	±6.37	0.921
Female										
Current smoking	Never	2879	(87.7)	2596	(87.9)	2587	(87.7)	2440	(88.6)	0.779
	Everyday	129	(3.9)	115	(3.9)	117	(4.0)	106	(3.9)	
	Sometimes	42	(1.3)	41	(1.4)	39	(1.3)	22	(0.8)	
	Past smoker	231	(7.0)	200	(6.8)	205	(7.0)	185	(6.7)	
Cigarettes per day	Never	3110	(94.7)	2796	(94.7)	2792	(94.7)	2625	(95.3)	
	(continuous)	8.89	±5.49	7.65	±5.24	8.29	±6.23	9.90	±6.00	0.010^1)^
Current smoking: electronic cigarettes	Never	3191	(97.2)	2876	(97.4)	2873	(97.4)	2668	(96.9)	0.048
	Everyday	20	(0.6)	21	(0.7)	23	(0.8)	29	(1.1)	
	Sometimes	22	(0.7)	5	(0.2)	7	(0.2)	12	(0.4)	
	Past smoker	48	(1.5)	50	(1.7)	45	(1.5)	44	(1.6)	
Electronic cigarettes per day	Never	3191	(97.2)	2876	(97.4)	2873	(97.4)	2668	(96.9)	
	(continuous)	6.45	±4.49	9.27	±8.01	7.2	±4.45	8.17	±5.93	0.222

n (%), the χ2 test and ANOVA has been performed.

1) Scheffe analysis was conducted: 2019 = 2021 = 2022 > 2020.

E-cigarettes, like the beginning of the year, saw an increase in the proportion of non-smokers during the COVID-19 period, and again in the post-COVID period. The group of men who used e-cigarettes daily decreased during the pandemic and increased to 5.45% in 2022 compared to 5.05% in 2019, while the number of women who used e-cigarettes gradually increased from 0.61% in 2019 to 1.05% in 2022. There was no statistically significant difference in e-cigarette smoking between men and women (Table 5).

### 4.4. Sleep and mental health

Weekday sleep time increased in the pre-COVID-19 and early COVID periods, and decreased again in the post-COVID period. Both men and women had the same results and were statistically significant. Weekend sleep time increased slightly in the With COVID-19 and post-COVID periods compared to the pre-COVID-19 and early COVID periods, and the same pattern was seen for both men and women. Stress was slightly reduced in the pre-COVID and post-COVID periods compared to the pre-COVID-19 and early COVID periods in men, and was statistically significant. In the case of women, stress has decreased in the post-COVID era, and the absolute number is higher than that of men (Table 6).

**Table 6:** Sleep and Mental health of the participants categorized by the respective year.

Variable		2019(n = 5,875)		2020(n = 5,379)		2021(n = 5,277)		2022(n = 4,844)		*P*-value
Sleep time(weekday)	(continuous)	6.63	±1.34	6.61	±1.38	7.02	±1.40	6.75	±1.30	0.000^1)^
Sleep time(weekend)	(continuous)	7.29	±1.71	7.24	±1.72	7.57	±1.53	7.50	±1.61	0.000^2)^
Stress	(continuous)	2.15	±0.73	2.17	±0.74	2.13	±0.74	2.09	±0.74	0.000^3)^
Suicidal ideation	Yes	72	(1.2)	100	(1.9)	69	(1.3)	50	(1.0)	0.002
	No	5803	(98.7)	5279	(98.1)	5208	(98.6)	4794	(98.9)	
Suicide attempts	Yes	24	(0.4)	25	(0.5)	26	(0.5)	23	(0.5)	0.921
	No	5851	(99.5)	5354	(99.5)	5251	(99.5)	4821	(99.5)	
Psychological counseling	Yes	178	(3.0)	189	(3.5)	196	(3.7)	217	(4.5)	0.001
	No	5697	(96.9)	5190	(96.4)	5081	(96.2)	4627	(95.5)	
Male										
Sleep time(weekday)	(continuous)	6.62	±1.27	6.61	±1.33	7.00	±1.41	6.80	±1.23	0.000^1)^
Sleep time(weekend)	(continuous)	7.38	±1.66	7.29	±1.64	7.58	±1.53	7.55	±1.56	0.000^2)^
Stress	(continuous)	2.12	±0.71	2.13	±0.73	2.08	±0.71	2.04	±0.72	0.000^3)^
Suicidal ideation	Yes	34	(1.3)	41	(1.7)	31	(1.3)	15	(0.8)	0.986
	No	2560	(98.6)	2386	(98.3)	2298	(98.6)	2076	(99.2)	
Suicide attempts	Yes	24	(0.4)	25	(0.5)	26	(0.5)	23	(0.5)	0.921
	No	5851	(99.5)	5354	(99.5)	5251	(99.5)	4821	(99.5)	
Psychological counseling	Yes	62	(2.4)	61	(2.5)	67	(2.9)	60	(2.9)	0.635
	No	2532	(97.6)	2366	(97.4)	2262	(97.1)	2031	(97.1)	
Female										
Sleep time(weekday)	(continuous)	6.63	±1.40	6.61	±1.43	7.03	±1.40	6.71	±1.34	0.000^1)^
Sleep time(weekend)	(continuous)	7.22	±1.74	7.20	±1.78	7.57	±1.53	7.46	±1.65	0.000^2)^
Stress	(continuous)	2.18	±0.75	2.20	±0.75	2.17	±0.76	2.14	±0.75	0.009^4)^
Suicidal ideation	Yes	38	(1.2)	59	(2.0)	38	(1.3)	35	(1.3)	0.024
	No	3243	(98.8)	2893	(98.0)	2910	(98.7)	2718	(98.7)	
Suicide attempts	Yes	15	(0.5)	15	(0.5)	17	(0.6)	15	(0.5)	0.924
	No	3266	(99.5)	2937	(99.5)	2931	(99.4)	2738	(99.5)	
Psychological counseling	Yes	116	(3.5)	128	(4.3)	129	(4.4)	157	(5.7)	0.001
	No	3165	(96.5)	2824	(95.7)	2819	(95.6)	2596	(94.3)	

n (%), the χ2 test and ANOVA has been performed.

1) Scheffe analysis was conducted: 2019 = 2020 < 2022 < 2021.

2) Scheffe analysis was conducted: 2019 = 2020 < 2021 = 2022.

3) Scheffe analysis was conducted: 2019 = 2020 > 2021 = 2022.

4) Scheffe analysis was conducted: 2019 = 2020 = 2021 > 2022.

For men, the proportion of 1 ~ 2 cups was higher, and for women, the proportion of 7 ~ 9 cups was higher, showing a difference. Binge drinking rates were higher in absolute numbers for men than for women, but in the time-wise variation, they gradually decreased for men and increased for women in groups almost daily. Drinking problems decreased during the pandemic but increased again in the post-COVID period, with the same results in the gender analysis (Table 6).

### 4.5. Difference in health behaviors and mental health by year

As a result of a logistic regression analysis that adjusted for the sociodemographic variables (Table 7), Frequency of drinking decreased in 2020 and 2021 for both men and women, but there was no difference in 2022. There was no statistically significant difference in current smoking for both men and women. Suicidal ideation decreased in men in 2022 (B = −0.65, p < 0.05), but increased in women during the early Corona period (B = 0.55, p < 0.05).

**Table 7:** Difference in drinking, smoking, and suicidal ideation by year.

	Frequency of drinking^1)^				Current smoking^2)^				Suicidal ideation^3)^			
	Male		Female		Male		Female		Male		Female	
	B(S.E)	*p*	B(S.E)	*p*	B(S.E)	*p*	B(S.E)	*p*	B(S.E)	*p*	B(S.E)	*p*
2020	−0.17(0.08)	0.023	−0.12(0.06)	0.038	−0.09(0.07)	0.204	−0.03(0.08)	0.743	0.24(0.24)	0.318	0.55(0.21)	0.009
2021	−0.30(0.08)	0.000	−0.15(0.06)	0.011	−0.13(0.07)	0.053	0.04(0.08)	0.635	−0.2(0.25)	0.935	0.06(0.23)	0.807
2022	−0.10(0.08)	0.224	0.02(0.06)	0.685	−0.07(0.07)	0.348	−0.05(0.08)	0.574	−0.65(0.31)	0.039	0.08(0.24)	0.736

* age, household income, educational level, type of residence, and region of residence were adjusted.

1) The frequency of drinking was dichotomized, with 0 representing ‘never’ and 1 representing ‘any drinking behavior.’

2) The current smoking was dichotomized, with 0 representing ‘never’ and 1 representing ‘any smoking behavior.’

3) The suicidal ideation was dichotomized, with 0 representing ‘yes’ and 1 representing ‘no’.

## 5. Discussion

This study aimed to examine suicidal ideation and attempt in adults aged 19 years and order using the 2019 ~ 2022 KCHS data and analyze the risk health behaviors changes pre, during, and post the COVID-19 pandemic. This study focused on identifying the health risk behaviors and mental health impacts of COVID-19 on men and women. Interpreting the results of the study from the social, economic, and psychological perspectives of the policies to prevent the spread of infectious diseases implemented in Korea during COVID-19 is meaningful in that it can prepare a national response plan in case of future [10].

Countries that have implemented national lockdown policies have reported an increase in the frequency and amount of alcohol consumed due to restrictions on social interaction [11–13]. Most studies report an increase in alcohol consumption [11–14] and South Korea saw a decline in alcohol consumption during the pandemic [15,16]. Since December 23, 2020 until July 2021, the Korean government’s ban on private gatherings of five or more individuals due to fear of infection, reduced social gatherings likely curtailed opportunities for social drinking, and thus the frequency of drinking has decreased significantly. Additionally, heightened anxiety, financial instability, and employment insecurity may have discouraged alcohol use in some segments of the population [17]. In particular, the decrease in the amount of alcohol consumed was more in men than in women, and the proportion of women who consumed 7 ~ 9 drinks, which corresponds to the criteria for high-risk drinking, increased. It should be noted that the proportion of those who consumed more than 10 cups did not change significantly. A study comparing 2019 ~ 2020 reported an increase in high-risk drinking among men [18], and the results of this long-term follow-up study showed an increase in the amount of alcohol consumption and frequency of binge drinking among women, suggesting that relaxation of social restrictions may have re-enabled risk behaviors, and that women may be differentially vulnerable to the long-term mental health sequelae of the pandemic.

In the same way that previous studies showed a decrease in smoking rates during the pandemic, male smokers also decreased in this study [18,19]. Some studies have reported an increase in smoking [15,20], and some studies reported a similar proportion of those who increased and decreased smoking [16]. Prior studies report that the increase in smoking volume was used as a relief from increased anxiety and stress in individuals during COVID-19. The decrease in smoking volume is believed to be a psychological effect of people to reduce the risk of health harm due to the high association between COVID-19 severity and smoking [16]. However, in this study, while male smoking rates declined throughout the pandemic, female smoking including both conventional and electronic cigarettes [21] increased slightly. During the COVID-19 period, there was an increase in the behavior of people who felt anxious about taking off their masks and smoking, so they quickly smoked in places where there were no people [21], and it is thought that the use of e-cigarettes, which are relatively less restrictive, has increased. This gender disparity may reflect different coping mechanisms or behavioral adaptations to pandemic-related stress. Collectively, prior literature suggests that smoking may serve as a maladaptive response to emotional distress, especially when alternative coping resources are limited. The uptick in e-cigarette use, particularly among young adults, may also reflect a shift toward more discreet and socially acceptable forms of nicotine consumption, especially under mask mandates or restrictions on traditional smoking environments [22].

The increase in suicidal ideation and psychiatric consultations, particularly among women, is also noteworthy. While men exhibited a decrease in suicidal ideation in 2022, women reported elevated levels in both early and post-pandemic periods. This may reflect gendered vulnerabilities during public health crises. Factors such as disproportionate caregiving responsibilities, greater exposure to job insecurity in service sectors, and increased incidence of domestic violence have been linked to elevated mental health risks among women [23]. These findings underscore the need for targeted mental health services and gender-sensitive intervention frameworks.

Most studies report an increase in sleep disturbances in the early stages of COVID [24,25]. A study in South Korea reported an increase in sleep duration during COVID-19, but a decrease in sleep satisfaction [26]. This result is due to changes in lifestyles due to the reduction in economic and social activities due to social distancing policies during the pandemic. Sleep is also highly associated with mental health, so strategies will be needed to improve it. Stress in the early days of COVID-19 was high, with similar results to previous studies [2,4,8]. From the results of this study, it is found that stress has improved in the with COVID-19 and post-COVID-19 periods compared to the early stage of COVID-19, indicating that the country is entering the stage of social recovery after COVID-19. However, in the case of women, the rate of suicidal ideation and psychiatric consultations increased significantly after the COVID-19 period, indicating that there are mental health problems, and it is necessary to identify factors that affect them in addition to COVID-19.

The strength of this study is that it follows and reports on health behaviors and mental health patterns in Korea in the pre-COVID-19, early-COVID, with-COVID, and post-COVID periods. In particular, it is different from previous studies that only compared the changes between 2019 ~ 2020, and it is a study that suggests the need for the Korean government’s intervention by reporting that the improved health behavior pattern due to the government’s social distancing policy during the COVID-19 period led to an increase in unhealthy behaviors again in 2022. In addition, the results of the study are representative because of the use of data from the National Health and Nutrition Examination Survey (KNHANES), which collects a nationwide sample.

There are limitations to this study. First, this study is a cross-sectional design, which limits the statistical power of estimating causal conclusions. This should be done by correcting for the time effect through the design of the panel model, which is the longitudinal data. Although the age distribution was generally balanced, the slightly higher proportion of older adults and lower proportion of younger adults reflects Korea’s aging population and the KNHANES sampling framework [23,27]. High-risk or severely ill individuals, particularly those experiencing mobility restrictions or mental health challenges, may have been underrepresented due to pandemic-related concerns about in-person participation [23,27]. This demographic skew may influence the interpretation of overall trends, as health behaviors can vary significantly by age. In addition, the sleep and mental health variables used in this study may be insufficient to fully capture qualitative aspects of mental health. Due to the limited range of variables available in the long-term dataset from 2019 to 2022, the present study was not able to explore in depth the complex interactions between health behaviors and mental health. It is therefore recommended that future research incorporate variables that better reflect qualitative dimensions, enabling a more nuanced understanding of these relationships.

## 6. Conclusion

During the COVID-19, the Korean government was relatively successful in overcoming social distancing as an appropriate intervention, but the country experienced major changes such as a decline in economic growth, a contraction in industry, a decrease in social activities, and an increase in unemployment, which had a significant impact on people’s health and mental health. Drinking and smoking showed a somewhat positive pattern of behavior change, but in the post-COVID-19, unhealthy behaviors have increased again, requiring active health interventions.

## References

[1] Korea Disease Control Prevention Agency. COVID-19virus disease-19 (COVID-19) [Internet]. Korea Disease Control Prevention Agency; 2024. [updated 2024 Feb 29]. Available from: https://www.k-stat.go.kr/metasvc/msba100/statsdcdta?statsConfmNo=117002

[2] Choi S, Kim H, Park J. The psychological impacts of COVID-19 on the general population and its association with health behaviors: A nationwide study in Korea. Psychiatry Research. 2021;295:113560.33187723

[3] Lee K, Kim Y, Cho S. Social isolation and mental health during the COVID-19 pandemic in Korea: Longitudinal analysis and policy implications. Korean Journal of Social Welfare. 2022;74(1):45–62.

[4] Lee J, Lee H, Lee M. Impact of COVID-19 pandemic on alcohol consumption and smoking behavior in the Korean population. Journal of Public Health. 2020;42(4):661–9.

[5] Shin S, Choi W. Effects of the COVID-19 pandemic on health behaviors and mental health in Korea. Psychology, Health & Medicine. 2021;26(4):467–74.

[6] Tibubos AN, Otten D, Ernst M, Beutel ME. A Systematic Review on Sex- and Gender-Sensitive Research in Public Mental Health During the First Wave of the COVID-19 Crisis. Front Psychiatry. 2021;12:712492. doi: 10.3389/fpsyt.2021.712492 34603104 PMC8484908

[7] Jeong H, Yim HW, Song YJ, Lee H. Changes in mental health during the COVID-19 pandemic: A review of studies in Korea. Journal of Korean Medical Science. 2021;36(42):e297.

[8] Yim H, Kim H, Choi K. The psychological and behavioral consequences of COVID-19 on Korean adults: A focus on alcohol consumption and smoking. Addiction. 2021;116(5):1091–7.

[9] Kim J, Lee H, Park S. A study on the early response to COVID-19 in Daegu, Korea, and the policy implications for epidemic control. Journal of Preventive Medicine and Public Health. 2020;53(5):267–73.

[10] Choi E-H. Health and health behavior changes in Korea before and during the COVID19 pandemic: KNHANES data analysis of the 2019~2020. The Korean Data & Information Science Society. 2022;33(6):1113–24. doi: 10.7465/jkdi.2022.33.6.1113

[11] Garnett C, Jackson S, Oldham M, Brown J, Steptoe A, Fancourt D. Factors associated with drinking behaviour during COVID-19 social distancing and lockdown among adults in the UK. Drug Alcohol Depend. 2021;219:108461. doi: 10.1016/j.drugalcdep.2020.108461 33454159 PMC7807168

[12] Koopmann A, Georgiadou E, Reinhard I, Müller A, Lemenager T, Kiefer F, et al. The Effects of the Lockdown during the COVID-19 Pandemic on Alcohol and Tobacco Consumption Behavior in Germany. Eur Addict Res. 2021;27(4):242–56. doi: 10.1159/000515438 33902030 PMC8247814

[13] Schmits E, Glowacz F. Changes in Alcohol Use During the COVID-19 Pandemic: Impact of the Lockdown Conditions and Mental Health Factors. Int J Ment Health Addict. 2022;20(2):1147–58. doi: 10.1007/s11469-020-00432-8 33424513 PMC7781407

[14] Barbosa C, Cowell AJ, Dowd WN. Alcohol Consumption in Response to the COVID-19 Pandemic in the United States. J Addict Med. 2021;15(4):341–4. doi: 10.1097/ADM.0000000000000767 33105169 PMC8327759

[15] Kang E, Lee H, Sohn JH, Yun J, Lee JY, Hong Y-C. Impact of the COVID-19 Pandemic on the Health Status and Behaviors of Adults in Korea: National Cross-sectional Web-Based Self-report Survey. JMIR Public Health Surveill. 2021;7(11):e31635. doi: 10.2196/31635 34653017 PMC8629347

[16] Choi S, Bahk J, Park S, Oh K, Jung-Choi K. Smoking, drinking, and physical activity among Korean adults before and during the COVID-19 pandemic: a special report of the 2020. 2022. 10.4178/epih.e2022043 PMC9133597 35538697

[17] Garnett C, Jackson S, Oldham M, Brown J, Steptoe A, Fancourt D. Factors associated with drinking behaviour during COVID-19 social distancing and lockdown among adults in the UK. Drug Alcohol Depend. 2021;219:108461. doi: 10.1016/j.drugalcdep.2020.108461 33454159 PMC7807168

[18] Sarich P, Cabasag CJ, Liebermann E, Vaneckova P, Carle C, Hughes S, et al. Tobacco smoking changes during the first pre-vaccination phases of the COVID-19 pandemic: A systematic review and meta-analysis. EClinicalMedicine. 2022;47:101375. doi: 10.1016/j.eclinm.2022.101375 35434579 PMC9002019

[19] Almeda N, Gómez-Gómez I. The Impact of the COVID-19 Pandemic on Smoking Consumption: A Systematic Review of Longitudinal Studies. Front Psychiatry. 2022;13:941575. doi: 10.3389/fpsyt.2022.941575 35903638 PMC9320170

[20] Patanavanich R, Glantz SA. Smoking Is Associated With COVID-19 Progression: A Meta-analysis. Nicotine Tob Res. 2020;22(9):1653–6. doi: 10.1093/ntr/ntaa082 32399563 PMC7239135

[21] Hwang J, Chun H-R, Cheon E. A qualitative study on the impact of COVID-19 on the behavior and attitudes of smokers and non-smokers in South Korea. BMC Public Health. 2021;21(1):1972. doi: 10.1186/s12889-021-12079-8 34724927 PMC8559696

[22] Hwang J, Chun HR, Cheon E. Impact of COVID-19 on smoker behavior in South Korea. BMC Public Health. 2021;21:1–8. 34724927 10.1186/s12889-021-12079-8 PMC8559696

[23] Kweon S, Kim Y, Jang M, Kim Y, Kim K, Choi S, et al. Data resource profile: the Korea National Health and Nutrition Examination Survey (KNHANES). Int J Epidemiol. 2014;43(1):69–77. doi: 10.1093/ije/dyt228 24585853 PMC3937975

[24] Pérez-Carbonell L, Meurling IJ, Wassermann D, Gnoni V, Leschziner G, Weighall A, et al. Impact of the novel coronavirus (COVID-19) pandemic on sleep. J Thorac Dis. 2020;12(Suppl 2):S163–75. doi: 10.21037/jtd-cus-2020-015 33214921 PMC7642637

[25] Alzueta E, Perrin PB, Yuksel D, Ramos-Usuga D, Kiss O, Iacovides S, et al. An international study of post-COVID sleep health. Sleep Health. 2022;8(6):684–90. doi: 10.1016/j.sleh.2022.06.011 36163137 PMC9501615

[26] Park K-H, Kim A-R, Yang M-A, Lim S-J, Park J-H. Impact of the COVID-19 pandemic on the lifestyle, mental health, and quality of life of adults in South Korea. PLoS One. 2021;16(2):e0247970. doi: 10.1371/journal.pone.0247970 33635897 PMC7909697

[27] Park JH, Lee J, Lee HJ. Potential bias in the Korea National Health and Nutrition Examination Survey and implications for interpreting trends. Epidemiology and Health. 2023;45:e2023023.

